# CD3^-^ Leukocytes Present in the Human Uterus During Early Placentation: Phenotypic and Morphologic Characterization of the CD56^++^ Population

**DOI:** 10.1155/1991/83493

**Published:** 1991

**Authors:** Ashley King, Nalini Balendran, P. Wooding, N. P. Carter, Y. W. Loke

**Affiliations:** 1 Division of Cellular and Genetic Pathology Department of Pathology University of Cambridge Tennis Court Road Cambridge CB2 1QP United Kingdom; 2 Institute of Animal Physiology and Genetics Research Cambridg Research Station Babraham Hall Cambridge CB2 4AT United Kingdom

## Abstract

In this study, the CD3- LGL/NK cells present in the pregnant human uterus have been characterized. Phenotypic and morphologic analyses of decidual LGL revealed many similarities to the minor CD56^bright+^, CD16^-^ subset in peripheral blood, but there were some important differences. The relative surface density of CD56^+^ is greatly increased on decidual LGL to 22 x that found on the majority of CD56 peripheral blood NK cells. The CD56bright cells in decidua show LGL morphology, whereas in peripheral blood, they are .mainly agranular. Proliferation of CD56^+^ cells occurs predominantly during the nonpregnant secretory (luteal) phase, indicating these CD56^+^ uterine LGL do not migrate as terminally differentiated cells. The appearance of CD56 cells was examined at the ultrastructural level using immunoelectron microscopy. Cells with phenotypic characteristics of decidual LGL occur in a higher percentage (1.11%) in the peripheral blood of women of reproductive age than in men (0.66%). On the basis of these results, it is proposed that the CD56^bright+^ uterine leukocytes represent a distinctive, hormonally regulated subset possibly adapted to control human placentation.


*Developmental Immunology*, 1991, Vol. 1, pp. 169–190
Reprints available directly from the publisher
Photocopying permitted by license only

© 1991 Harwood Academic Publishers GmbH
Printed in the United Kingdom

### CD3⁻ Leukocytes Present in the Human Uterus During Early Placentation: Phenotypic and Morphologic Characterization of the CD56⁺⁺ Population

ASHLEY KING,*† NALINI BALENDRAN,† P. WOODING,§ N. P. CARTER† and Y. W. LOKE†

†*Division of Cellular and Genetic Pathology, Department of Pathology, University of Cambridge, Tennis Court Road, Cambridge CB2 1QP, United Kingdom*
§*Institute of Animal Physiology and Genetics Research, Cambridge Research Station, Babraham Hall, Cambridge CB2 4AT, United Kingdom*

In this study, the CD3⁻ LGL/NK cells present in the pregnant human uterus have been characterized. Phenotypic and morphologic analyses of decidual LGL revealed many similarities to the minor $CD56^{bright+}$, $CD16^-$ subset in peripheral blood, but there were some important differences. The relative surface density of $CD56^+$ is greatly increased on decidual LGL to 22× that found on the majority of $CD56^+$ peripheral blood NK cells. The $CD56^{bright+}$ cells in decidua show LGL morphology, whereas in peripheral blood, they are mainly agranular. Proliferation of $CD56^+$ cells occurs predominantly during the nonpregnant secretory (luteal) phase, indicating these $CD56^+$ uterine LGL do not migrate as terminally differentiated cells. The appearance of $CD56^+$ cells was examined at the ultrastructural level using immunoelectron microscopy. Cells with phenotypic characteristics of decidual LGL occur in a higher percentage (1.11%) in the peripheral blood of women of reproductive age than in men (0.66%). On the basis of these results, it is proposed that the $CD56^{bright+}$ uterine leukocytes represent a distinctive, hormonally regulated subset possibly adapted to control human placentation.

KEYWORDS: Large granular lymphocytes, uterus, $CD3^-$ cells, $CD56^+$ cells, pregnancy.

### INTRODUCTION

At the time the human placenta is established, the predominant leukocytes (>70%) present in the maternal uterine mucosa (decidua) are large granular lymphocytes (LGL) with a distinctive phenotype ($CD3^-$, $CD16^-$, $CD56^+$) and natural killer (NK) function (Starkey et al., 1988; Bulmer, 1989; King et al., 1989a, 1989b; Manaseki and Searle, 1989). There is a significant variation in the numbers of these cells during the menstrual cycle, indicating that their recruitment to the uterus is probably under hormonal control. However, it has not yet been established to what lineage these decidual LGL belong. In peripheral blood, a very small subset (<1%) of LGL appears to have similar phenotypic and functional characteristics (Lanier et al., 1986), but it is still unclear how they relate to decidual LGL. Recently, Lanier and his colleagues have made a comprehensive analysis of peripheral blood LGL/NK cells on the basis of both qualitative and quantitative expression of their cell-surface markers, together with morphological features such as granularity and the degree of NK cytolytic activity (Nagler et al., 1989). We now report a similar analysis of decidual LGL. In addition, we have studied these cells at the ultrastructural level using immunoelectron microscopy and have also compared the numbers of $CD3^-$, $CD16^-$, $CD56^{bright+}$ LGL in peripheral blood of males and females to see whether there are any sex differences. To assess whether $CD56^+$ LGL migrate to the uterus as terminally differentiated cells or whether they can proliferate *in situ*, we have used double immunohistological staining for the proliferation marker, Ki-67 combined with CD56 on frozen sections of pregnant and nonpregnant endometrium. Our findings lead us to conclude that decidual LGL may represent a unique uterine-specific lymphoid population.

*Corresponding author.

### RESULTS

### Characterization of CD56$^{bright+}$ Peripheral Blood Lymphocytes in Males and Females

Two-colour immunofluorescence of PBL with PE-conjugated-Leu-19 (CD56) plus Leu-11b (CD16) and Leu-4 (CD3) followed by FITC. GAM revealed three distinct subsets (Fig. 1) with a small population of CD3$^-$, CD16$^-$, CD56$^{bright+}$ cells (Fig. 1; Box R1), as previously described (Lanier et al., 1986). We also found that this subset comprised <2% of peripheral blood lymphocytes. The proportion of cells with this phenotype was compared in eight females aged 18–25 years and seven similarly aged males (Table 1). Histograms of a representative male and female are shown in Figs. 2a and 2b. It was found that the mean percentage of these cells in males (0.66% of total PBL) was significantly lower than in females (1.11%) (Student's $t$ test, $t$=3.43, $p$=<.005). The proportion of CD3$^-$, CD16$^-$, CD56$^{bright+}$ cells was analyzed in three females weekly throughout the menstrual cycle. Although there was a slightly lower proportion of these cells in the secretory (luteal) phase in two women, these differences were not significant (data not shown).

TABLE 1
Percentages of CD3$^-$, CD16$^-$, CD56$^{bright+}$ in PBL of Males and Females[a]

| Males (%) | Females (%) | Time in menstrual cycle |
|---|---|---|
| 0.52 | 1.51 | 1/28 |
| 0.74 | 1.05 | 6/28 |
| 0.61 | 0.94 | 1/28 |
| 0.68 | 0.57 | 17/28 |
| 0.78 | 0.78 | 27/28 |
| 0.61 | 1.31 | |
| 0.68 | 1.45 | 25/33 |
| | 1.43 | 1/28 |
| Mean = 0.66 ± 0.03 | Mean = 1.11 ± 0.11 | |

[a]Analysis was performed randomly throughout the menstrual cycle in females. The time in the cycle is given from the first day of the first menstrual period.

### Characterization of Decidual Leukocytes

*LCA.* Six preparations of decidual leukocytes, pooled from three to four pregnancies and isolated by mechanical sieving followed by separation over Lymphoprep, were stained for leukocyte common antigen (CD45) to assess the success of our extraction method for bone-marrow-derived cells. In these

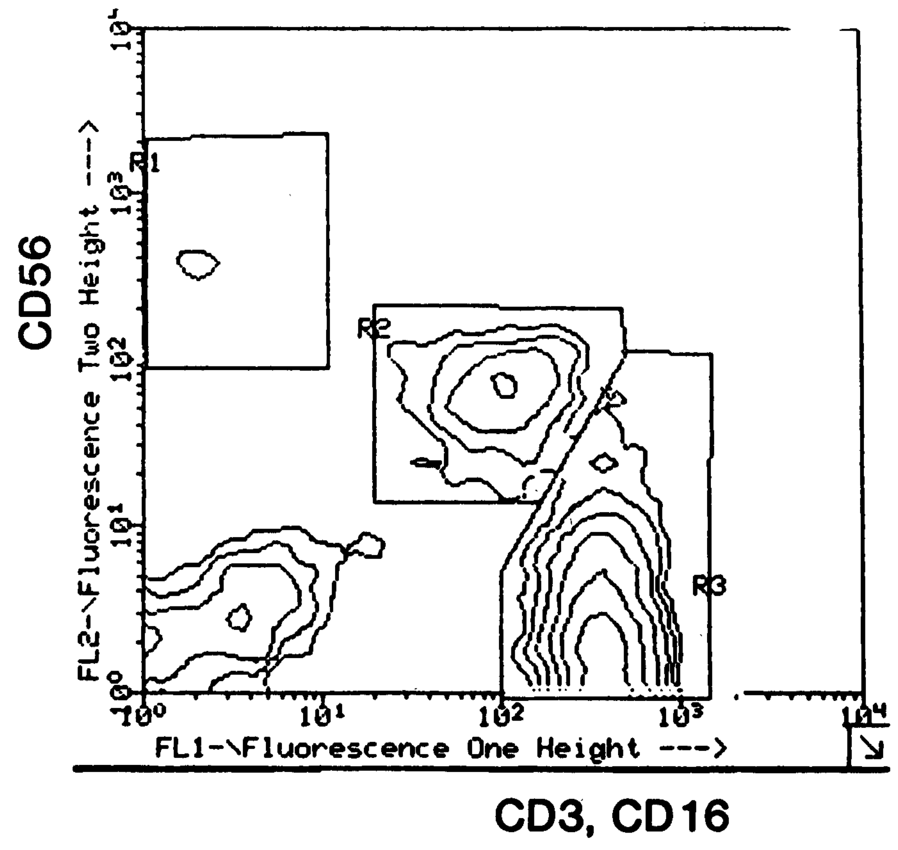

FIGURE 1. PBL were purified over Lymphoprep and stained with PE-conjugated anti-CD56 (Leu-19) followed by anti-CD3 (Leu-4) and anti-CD16 (Leu-11b) with a secondary FITC-conjugated antibody. A representative two-color contour map displaying PE (CD56) and FITC (CD3 and CD16) fluorescence. Three subsets were identified as indicated by boxes labeled R1–R3. R1: CD56$^{bright+}$, CD16$^-$, CD3$^-$. R2: CD56$^{dim+}$, CD16$^{dim+}$, CD3$^{dim+}$. R3: CD56$^-$, CD16$^+$CD3$^+$.

preparations of decidual cells, ~90% of cells were LCA$^+$ at the appropriate gate setting (Fig. 3).

*CD56*. The proportion of CD56$^+$ cells in 12 decidual cell preparations was analyzed. 70% ± 12% of cells were CD56$^+$. As in peripheral blood, the CD56$^+$ cells can be divided into CD56$^{bright+}$ and CD56$^{dim+}$ subsets (Fig. 2c) on the basis of fluorescence intensity. The majority (80%) are CD56$^{bright+}$. Lanier has shown that the CD56$^{bright+}$ PBL population expresses ~3.5 more Leu-19 antigen than the CD56$^{dim+}$ population (Lanier et al., 1986). We have extended this observation by comparing the decidual

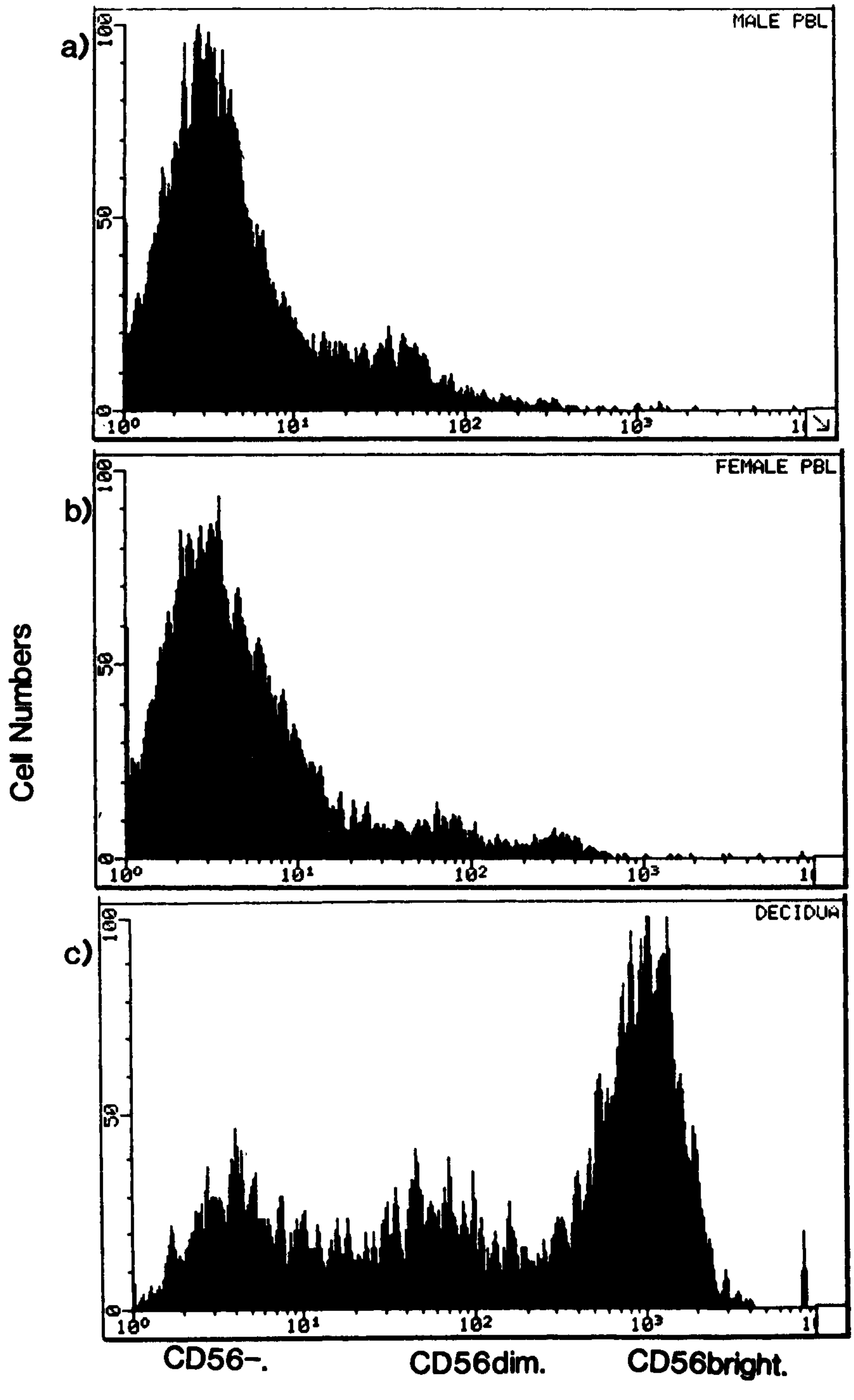

FIGURE 2. Representative single-color histogram of CD56$^+$ cells analyzed from peripheral blood of a (a) normal male donor, (b) normal female donor, and (c) decidua. In peripheral blood, the tiny CD56$^{bright+}$ population, larger CD56$^{dim+}$ population, and CD56$^-$ population are clearly seen. By contrast in decidual cell preparations, the majority of cells are CD56$^{bright+}$, with smaller CD56$^{dim+}$ and CD56$^-$ populations.

CD56$^+$ antigenic density levels to that of peripheral blood (Fig. 4). It was found that CD56 is ~5× brighter on CD56$^{bright+}$ PBL than CD56$^{dim+}$ PBL in males and ~6.4× brighter in females. In CD56$^+$ decidual cells, the antigenic density of CD56 is 22× that in CD56$^{dim+}$ PBL and ~4× brighter than CD56$^{bright+}$ PBL. In decidual LGL, the CD56$^{bright+}$ population is 14× brighter than CD56$^{dim++}$. After culturing in rIL-2 for 1-4 days, there was a further slight increase in the density of CD56.

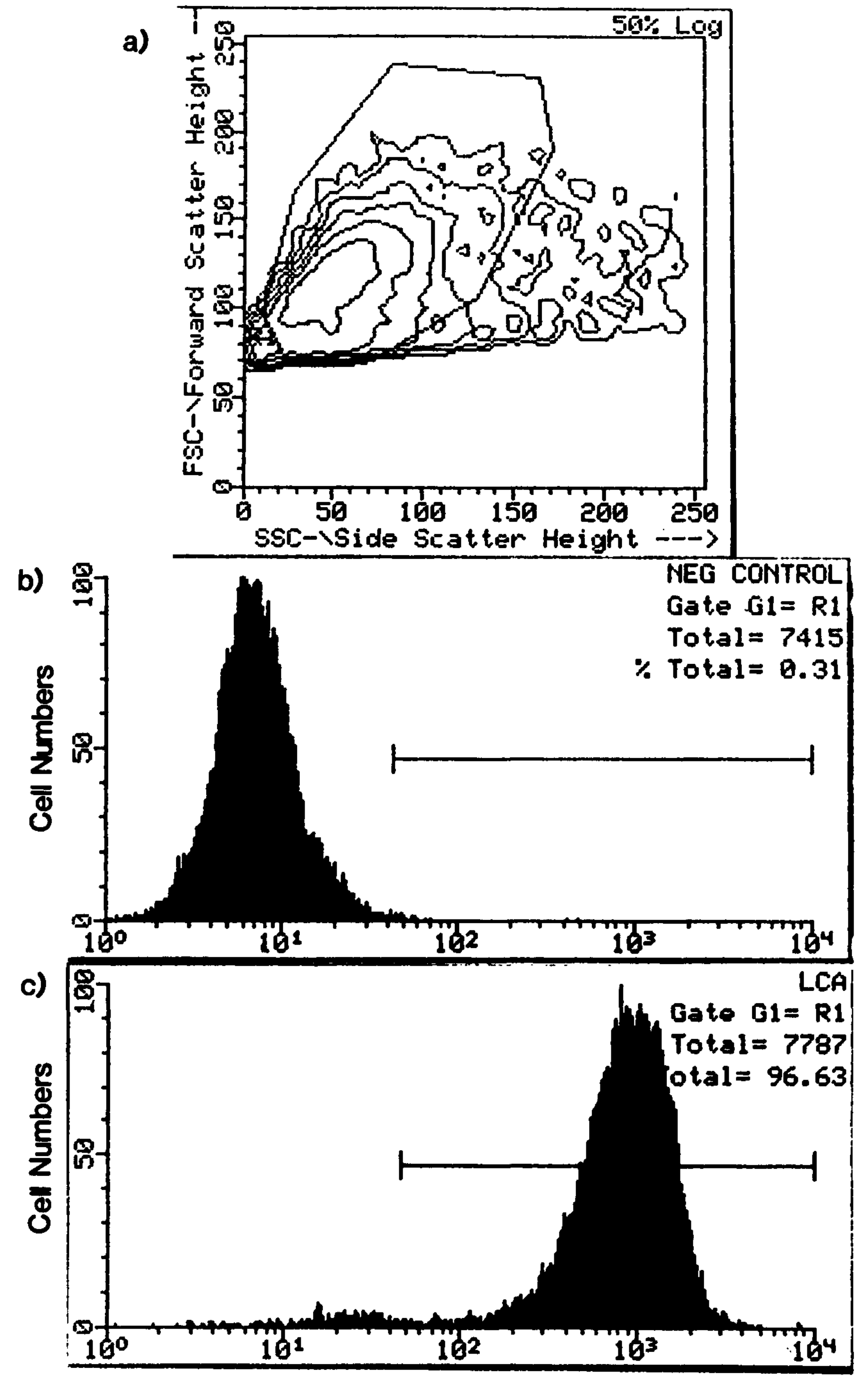

FIGURE 3. Decidual leukocytes extracted by mechanical sieving followed by centrifugation over Lymphoprep and staining with FITC-LCA (CD45). The cells were gated as shown (a). The negative control is seen in (b), and the proportion of CD45$^+$ positive cells in one such preparation is shown in (c). ~90% of cells in decidual cell preparations were CD45$^+$.

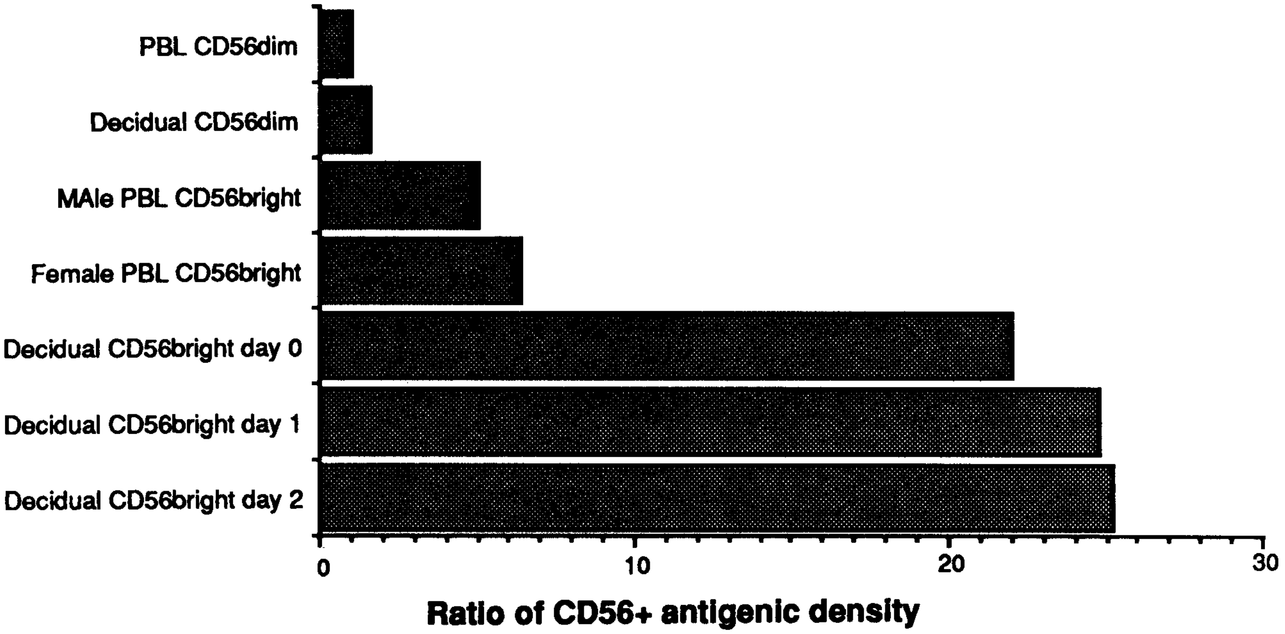

FIGURE 4. Relative expression of CD56 antigen as measured by linear flourescence in $CD56^+$ PBL and decidual cells.

*Morphology of CD56 Cells.* The morphology of $CD56^+$ cells was analyzed by forward and side scatter in both peripheral blood and decidua. The $CD56^{bright+}$ decidual cells appear larger and more granular than their peripheral blood counterparts (Figs 5a and 5b). The decidual $CD56^{dim+}$ subset was also granular (results not shown). One decidual cell preparation was sorted into $CD3^-$ and $CD56^{bright+}$ cells and cytospin smears of $CD56^{bright+}$ cells were stained with Giemsa. More than 75% of $CD56^{bright+}$ cells were observed to be granular (Fig. 6a) as opposed to 0.5–25% of $CD56^{bright+}$ cells in peripheral blood (Lanier et al., 1986).

### Phenotype of $CD56^{bright+}$ and $CD56^{dim+}$ cells

A detailed phenotypic characterization of $CD56^+$ decidual leukocytes was performed using two-color immunofluorecence. Each Mab was tested on at least three separate occasions. Live cells were analyzed and markers defining quadrants were positioned to exclude >99% of unstained cells in the lower left quadrant of a negative control sample, which included a minor autofluorescent population. Because there was a biphasic expression of the CD56 antigen, the data were also analyzed by setting regions around each distinct population. All the data shown were analyzed using the same fixed regions. This also allowed the fraction of autofluorescent cells, which may overlap with the weakly staining cells, to be subtracted from the results. These results are summarized as a histogram (Fig. 7) and contour diagrams (Fig. 8).

*T-Cell Markers.* It can be seen that the majority of $CD56^+$ decidual cells co-express CD2, CD7, CD45RA, CD11a, and CD18 like their peripheral blood counterparts, but not mature T-cell markers such as CD3 and CD5. We find there is a differential expression of CD2 and CD7 surface antigens on $CD56^{bright+}$ and $CD56^{dim+}$ decidual subsets, being stronger in the $CD56^{bright+}$ cells (results not shown). However, while most decidual $CD56^{bright+}$ cells are $CD2^+$ and $CD7^+$, there is a small population (5–10%) that is either $CD2^-$ or $CD7^-$. We have also observed an additional small subpopulation (~3%) of decidual cells that is $CD56^{bright+}$, and co-expresses the TCR-associated CD3 molecule. However, their size and granularity show they are large granulated cells unlike classical T cells (Fig. 9a).

*NK Cell Markers. a) CD57.* The NK marker CD57 is absent from most $CD56^+$ decidual cells. This is in accord with the findings of Nagler et al. (1989) that

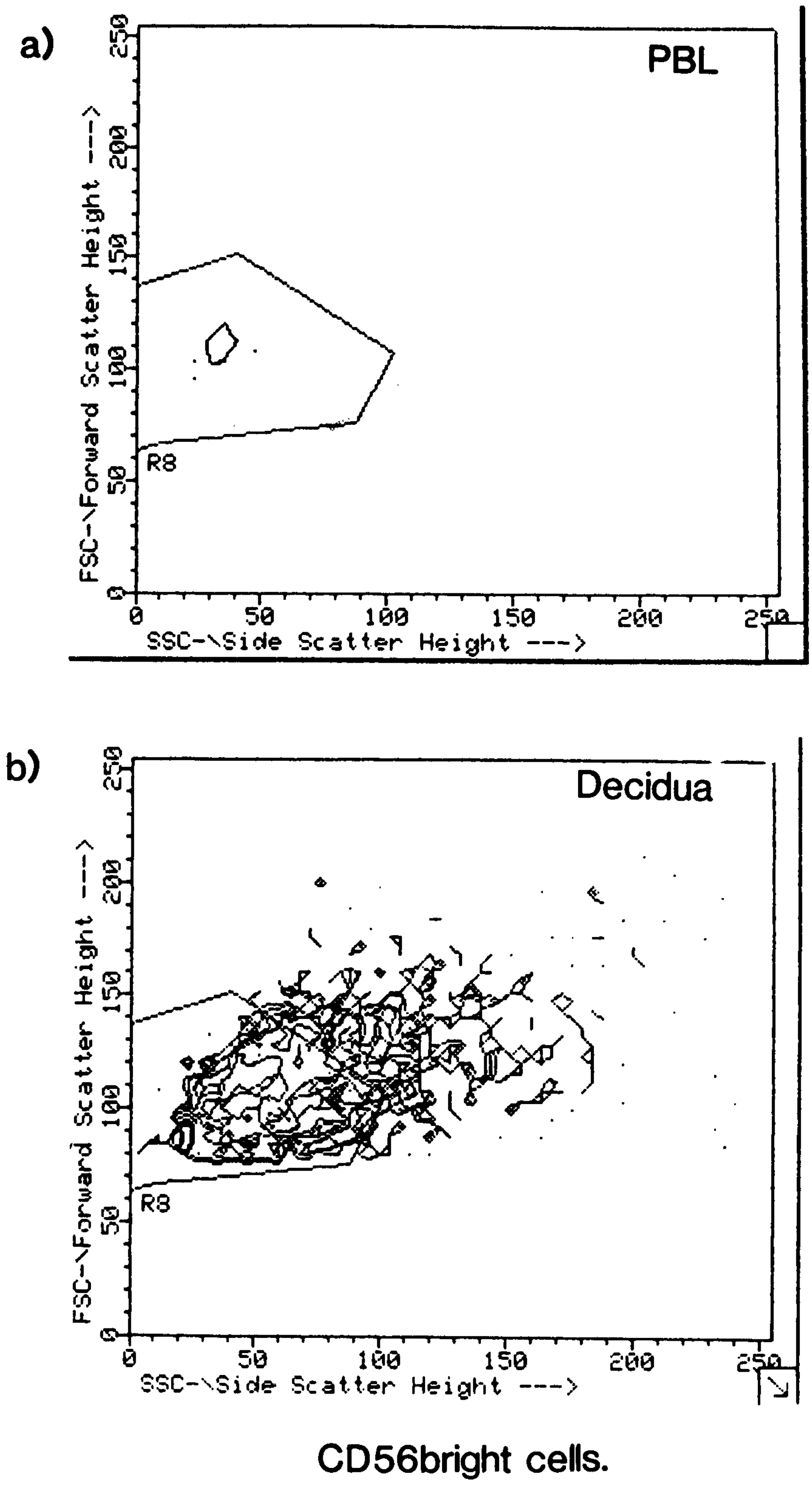

FIGURE 5. $CD56^{bright+}$, $CD16^-$, and $CD3^-$ cells analyzed by forward and side scatter in both (a) peripheral blood and (b) decidua. The inset indicates the size and granularity of normal peripheral blood lymphocytes. It is apparent that in decidua, the $CD56^{bright+}$ cells are larger and more granular.

CD57 was not expressed on PBL $CD56^{bright+}$, $CD16^{dim+}$/$CD16^{-}$ populations.

*b*) *CD16*. Three distinct NK cell subsets have been identified in peripheral blood on the basis of CD16 expression (Nagler et al., 1989): two small populations (<1%) of $CD56^{bright+}$ cells, and $CD16^{dim+}$ or $CD16^{-}$; and a major (10–15%) classical NK population $CD56^{dim+}$ $CD16^{bright+}$. We have identified both $CD16^{-}$ and $CD16^{dim+}$ in the $CD56^{+}$ decidual lymphocytes. The $CD56^{bright+}$ were almost entirely $CD16^{-}$ with only a very few cells expressing very low levels of CD16 (Fig. 10c). The small $CD56^{dim+}$ population was also $CD16^{dim+}$ (Fig. 10b). However, as this population overlaps with the minor autofluorescent population, some of the $CD16^{+}$ cells may be autofluorescent cells. We have allowed for this by gating round the regions and subtracting the autofluorescent population on the negative control and we still find that ~13% $CD56^{+}$ cells express low levels of CD16. CD16 is also expressed at low density on some $CD56^{-}$ cells (Fig. 10a). There appear, therefore, to be subsets of CD56 cells in decidua in relation to the level of expression of both CD56 and CD16.

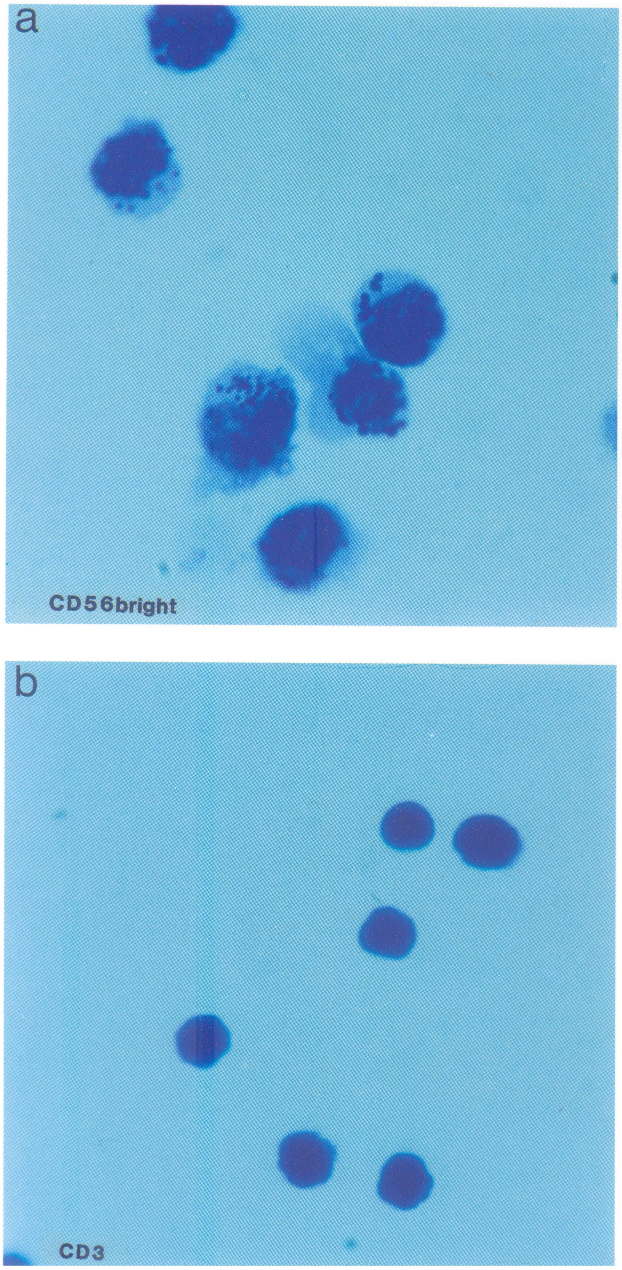

FIGURE 6. Cytospin smears of sorted (a) $CD56^{bright+}$ and (b) $CD3^{+}$ decidual cells stained with Giemsa. Numerous leukocytes with typical large granular lymphocyte morphology are seen in $CD56^{+}$ smears. The $CD3^{+}$ cells appear to be small agranular lymphocytes. (See Colour Plate VII at the back of this publication.)

*Activation Markers.* CD69 is detectable on the surface on NK cells within a few hours after exposure to IL-2, but reacts only weakly with resting peripheral blood NK cells (Lanier et al., 1988). We found 40% of freshly isolated $CD56^{+}$ decidual cells expressed this antigen, yet it was reported to be absent from $CD56^{+}$ PBL (Nagler et al., 1989).

HML-1 is an antigen detected on more than 90% of human intestinal intraepithelial lymphocytes and 40% of intestinal lamina propria lymphocytes, whereas in other tissues, it is rarely or not expressed (Bensussan et al., 1987). We confirm that this antigen is not confined to the unique environment of the gut, but is also detectable on 20% of both bright and dim subsets of $CD56^{+}$ decidual leukocytes.

### Other Leukocytes Present

1. *$CD3^{+}$ Cells.* A population of $CD56^{-}$, $CD3^{+}$ cells was always present in the decidual preparations amounting to ~10%. The morphology of these cells as assessed by foreward/side scatter (Fig. 9a) and Giemsa staining of sorted $CD3^{+}$, $CD56^{-}$ cells was of small agranular lymphocytes (Fig. 6b). They are likely to be mature T cells.

2. *Macrophages.* HLA $DR^{+}$ Leu $M3^{+}$ macrophages are a characteristic feature of early decidua (Bulmer, 1989), where they account for ~20% of cells in immunohistological studies (Bulmer et al., 1988;

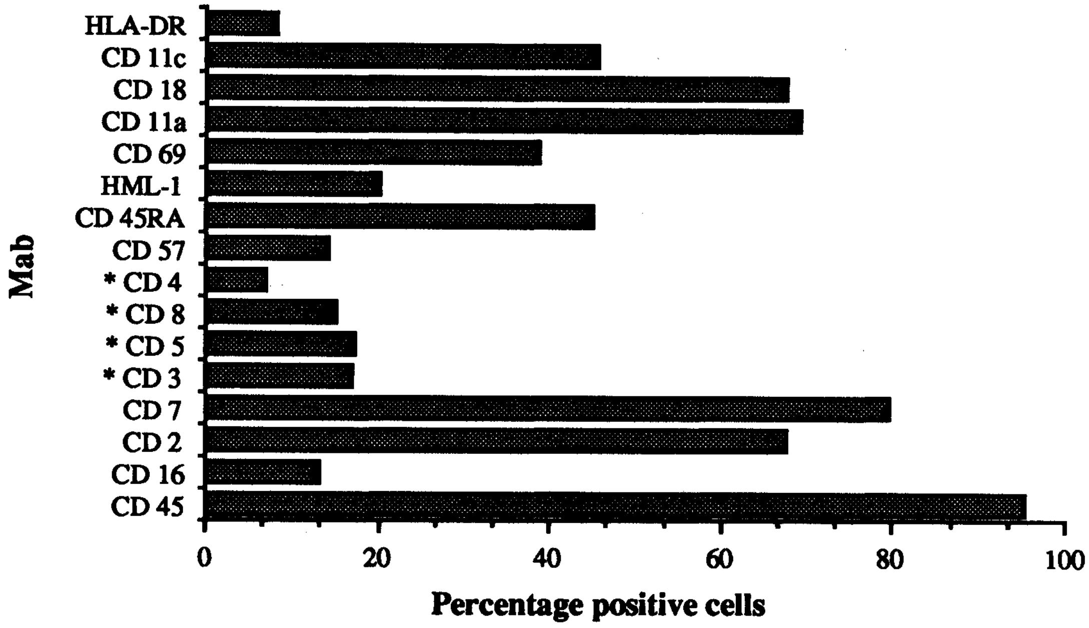

FIGURE 7. Leukocyte differentiation antigens expressed on decidual CD56⁺ cells. The results were analyzed using two-color fluorescence as described in Methods. The (*) indicates antigens expressed at very low density on CD56 cells.

King et al., 1989a). These cells comprised ~5–10% of our decidual preparations as assessed by HLA DR⁺ cells. The lower numbers detected may be due to the mechanical sieving procedure used to isolate LGL. LGL are probably more loosely attached to the stroma than macrophages and are, therefore, more easily extracted.

### Proliferation of CD56⁺ Lymphocytes *In Vivo*

Proliferating cells were identified using Ki-67, a Mab detecting a nuclear proliferation antigen present on all cells not in GO (Gerdes et al., 1984). The sections were subsequently stained with Leu-19 to determine whether proliferation of CD56⁺ cells occurred *in vivo*. The proportion of at least 200 CD56⁺ cells, which were also Ki-67⁺, was counted in endometrium at all stages of the menstrual cycle and in decidua from 6–16 weeks gestational age.

In proliferative-phase endometrium, numerous proliferating cells were found in both glands and stroma (Fig. 11a). CD56⁺ cells were sparse and Ki-67⁺ were also very few (Fig. 12). However, in the secretory phase, Ki-67⁺ cells were only found in the endometrial stroma apart from endothelial cells, which were often Ki-67⁺. Most Ki-67⁺ cells were also CD56⁺, indicating that it is the LGL and not stromal and glandular cells that were proliferating at this stage of the cycle. Specimens of decidua showed a similar picture, although, in general, there appeared to be fewer Ki-67⁺ cells in the stroma (Fig. 11b). The proportion of CD56⁺ cells that were also Ki-67⁺ is given in Fig. 12. It is apparent that the maximal proliferation occurs in the non-pregnant secretory-phase endometrium. Although some Ki-67⁺/CD56⁺ cells are seen in decidua, there were less proliferating CD56⁺ cells with a slightly downward trend as gestation proceeded. Decidua from a molar pregnancy also contained numerous CD56⁺ cells, of which a proportion was Ki-67⁺ similar to a normal pregnancy.

### Electron Microscopy

To compare the ultrastructural appearances of decidual LGL with that reported for both endometrial granulocytes (Cardell et al., 1969; Loubet et al., 1989) and NK/LGL in the peripheral blood (Kang et al., 1987; Polli et al., 1987), electron microscopy was performed together with immunoelectron microscopy for CD56.

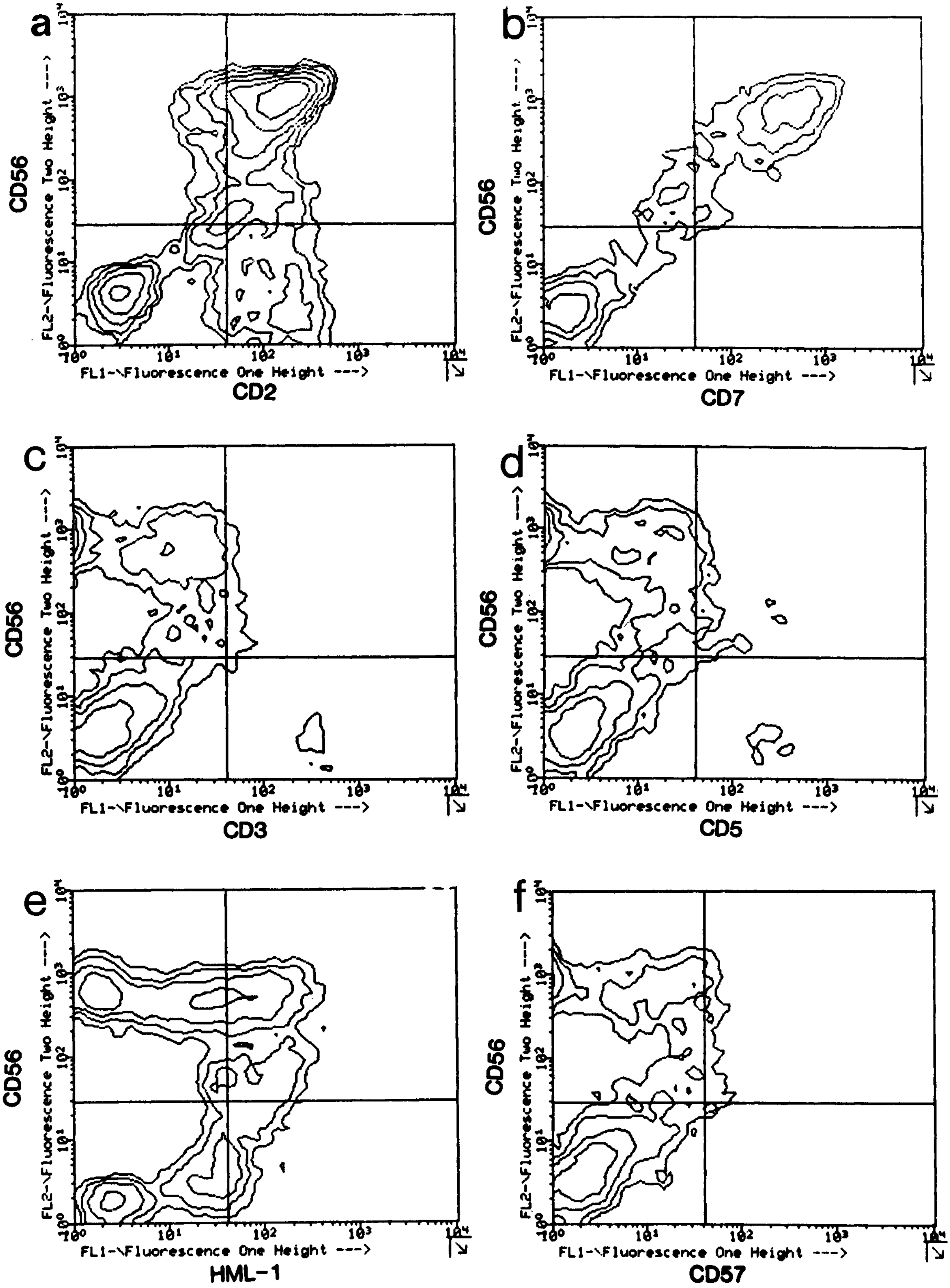

FIGURE 8. Two-color contour plots of CD56 (PE) versus FITC-conjugated (a) CD2, (b) CD7, (c) CD3, (d) CD5, (e) HML-1, and (f) CD57. CD56 cells obviously co-express CD2 and CD7, but only a few co-express low levels of CD3, CD5, and CD57. HML-1 is also present on some $CD56^{+}$ cells.

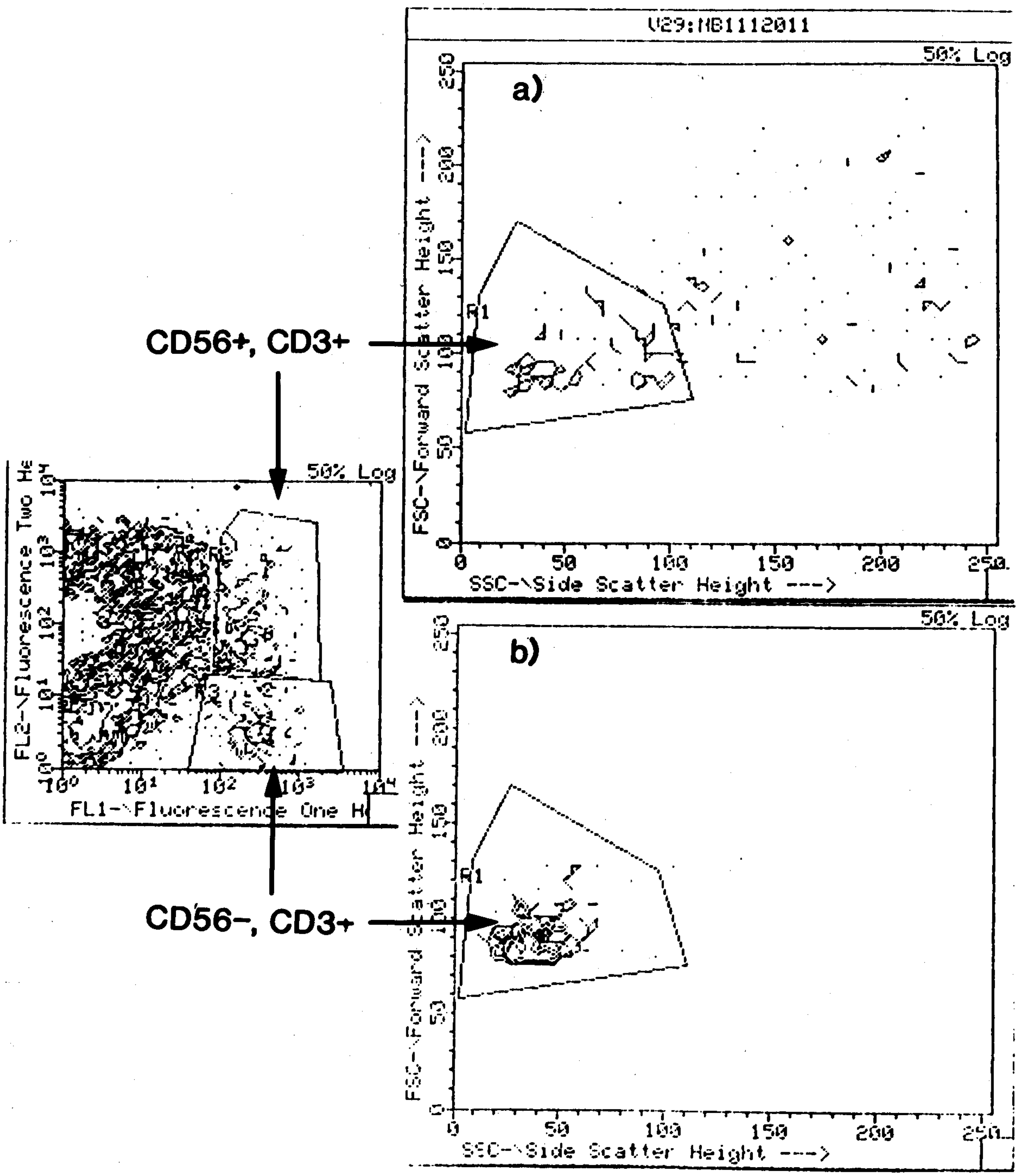

FIGURE 9. Size and granularity of $CD3^+$ subsets in decidual-cell preparations stained with PE-CD56 and FITC-CD3. (a) $CD3^+$, $CD56^+$, and (b) $CD3^+$, $CD56^-$ populations were gated and the morphology and granularity assessed by forward and side scatter. The size and granularity of the two $CD3^+$ populations is clearly different.

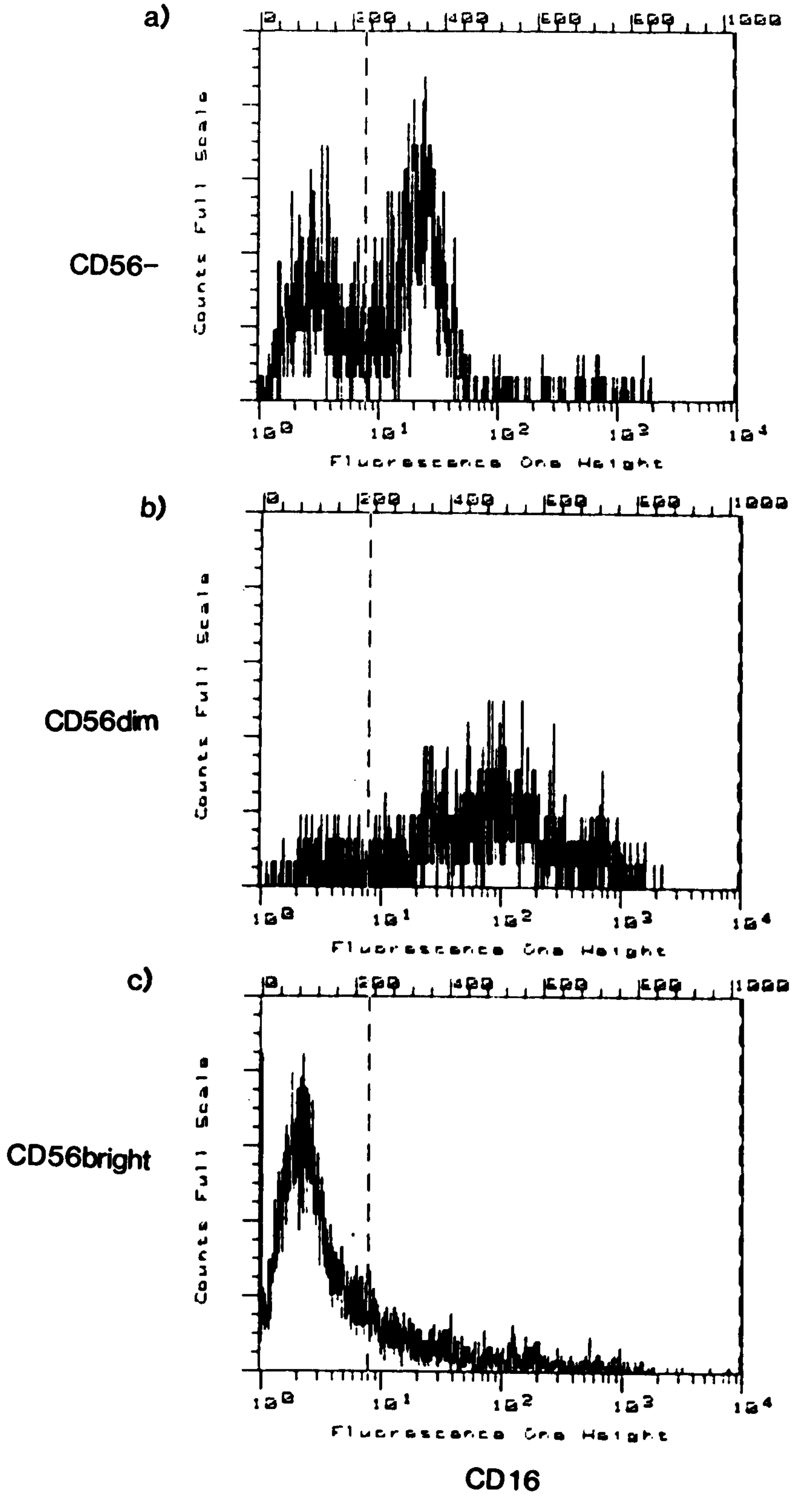

FIGURE 10. Representative single-color histogram of CD16$^+$ cells in (a) CD56$^-$ cells, (b) CD56$^{dim+}$, (c) CD56$^{bright+}$, as gated on a separate PE histogram (CD56$^+$) (as in Fig. 2c). The CD56$^{dim+}$ cells also include the minor autofluorescent population, as explained in the results.

### Morphology

Sections contained glands, uterine stromal cells, blood vessels, and leukocytes. Stromal cells were easily recognized by their large size, pale nucleus, and cytoplasm with evenly distributed organelles. Of the leukocytes, the most frequent were round 10–15 $\mu$m diameter cells containing a round or kidney-shaped nucleus with dense chromatin, small mitochondria, and a few elongated cisternae of rough endoplasmic reticulum (Fig. 13A). Their cytoplasm was much denser than that of the stromal cells and fairly short cytoplasmic projections were often seen. The most characteristic feature of these cells was the presence of 1–10 membrane-bound granules (0.2–0.4 $\mu$m) per section of cell. The granules mostly had a homogeneous dense content, but sometimes had a dense core surrounded by a variable-width light zone containing small vesicles, granular material and irregular membranous inclusions (Fig. 13B). No "paracrystalline" or "parallel tubular" arrays were found in the granule periphery. The Golgi body varied considerably in size; the large

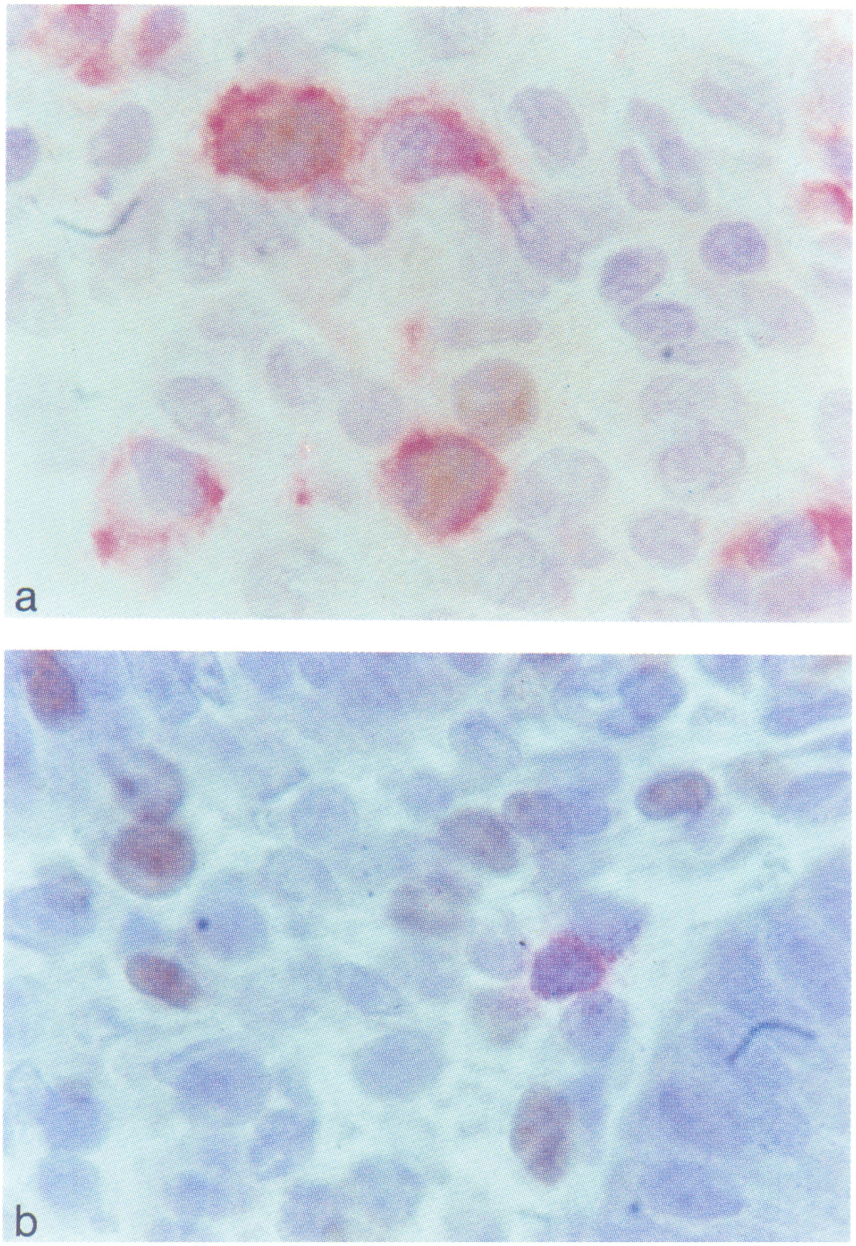

FIGURE 11. Frozen sections of (a) proliferative endometrium and (b) decidua (7 weeks gestation) double stained with the nuclear proliferation Mab, Ki-67 (brown), and CD56 (red). In the proliferative phase, nuclei are seen to be Ki-67$^+$ in both glandular and stromal cells distinct from the sparse CD56$^+$ cells. In early decidua, Ki-67$^+$ cells are also CD56$^+$, indicating the CD56$^+$ LGL are proliferating. (See Colour Plate VIII at the back of this publication.)

ones usually occurred at the centre of a group of granules. Dividing granulated leukocytes (Fig. 13C) and intraepithelial leukocytes containing the characteristic granules (Fig. 13D) were also observed. A significant number of decidual leukocytes appeared similar ultrastructurally but contain no granules. The macrophage was the only other leukocyte seen in significant numbers. Ultrastructurally, macrophages had a far more complex and divided outline. They contained granules, usually far smaller than those present in granulated leukocytes, but some were of similar dimensions and appearance. There were, therefore, cells present that were not classifiable as granulated lymphocytes or macrophages on ultrastructural grounds alone. The nature of the granulated leukocytes was therefore confirmed using immunoelectron microscopy to CD56.

### Immunoelectron microscopy

*Post-embedding Localization on Whole Tissue.* None of the techniques tried was successful on either Araldite or K4M-embedded sections of decidua. Neither Leu-19 or Leu-M3 produced any significant level of gold labeling, confirming previous reports that attempted this method (Kang et al., 1988).

*Pre-embedding Localization on Isolated Cells.* Incubating with Leu-19 prior to fixation and embedding resulted in labeling of the leukocyte plasmalemma. Using the single-sandwich technique resulted in a plasmalemma label of 2–3 gold particles per cell perimeter, which was barely above the background of 1–2 particles. However, the double-sandwich technique produced a higher, if somewhat variable, label between 30–100 particles per cell perimeter (Fig. 14). No significant labeling was seen in the cytoplasm. Decidual stromal cells, glandular cells, and endothelial cells never labeled above background levels. All cells showing the morphological attributes of uterine granulated lymphocytes labeled with Leu-19. The few unlabeled granulated leukocytes could morphologically equally well be classified as macrophages. A variety of leukocytes without granules labeled with Leu-19. Some were small and round with little cytoplasm; others were elongated. Since serial sections were not taken, it is possible that some of these cells did have granules and cytoplasmic inclusions out of the plane of the few sections examined per cell.

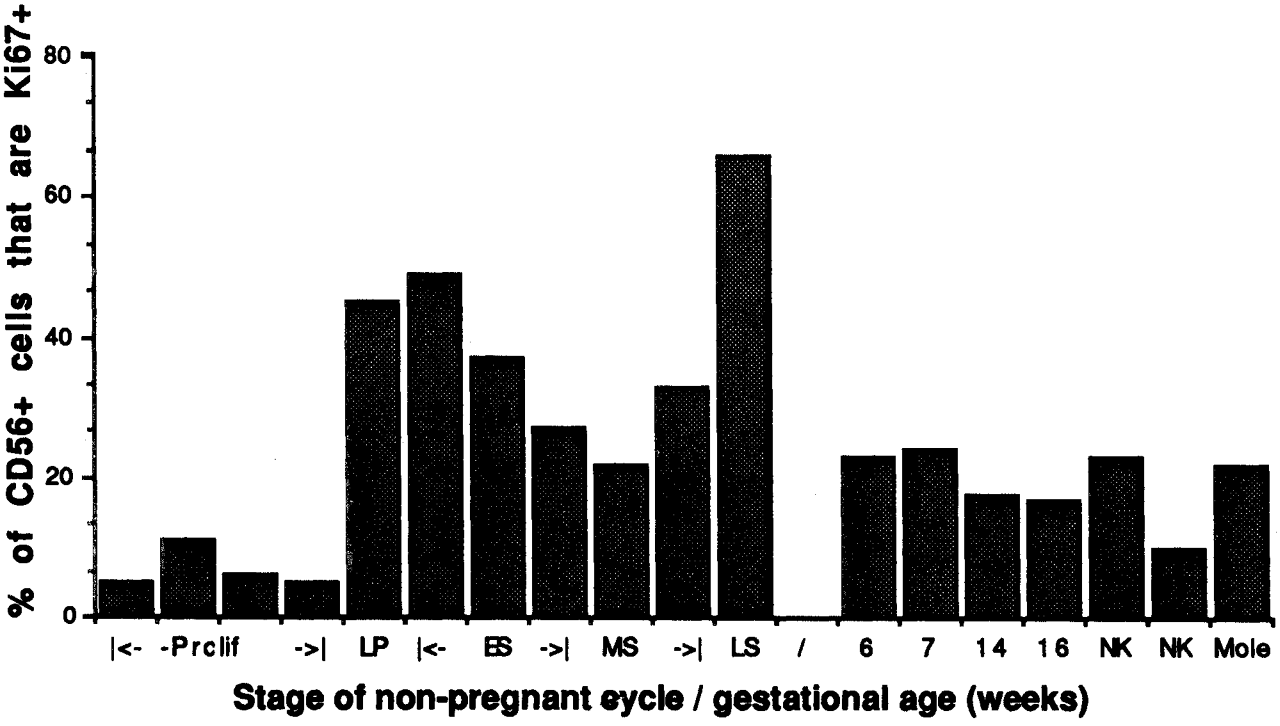

FIGURE 12. Proportion of $CD56^+$ cells in sections of endometrium and decidua that were also $Ki\text{-}67^+$.

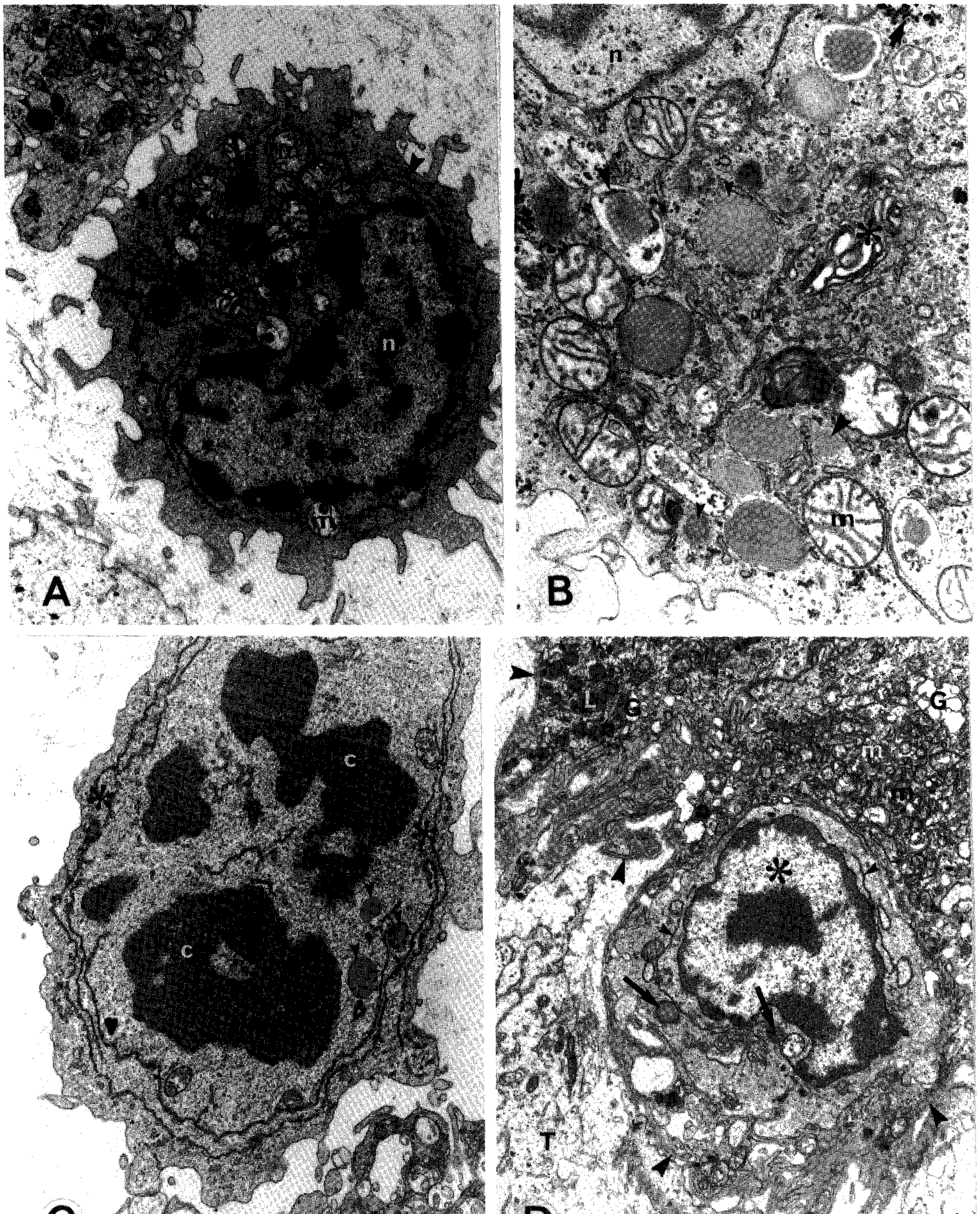

A
n
B
n
m
C
c
c
D
L
G
G
m
m
T

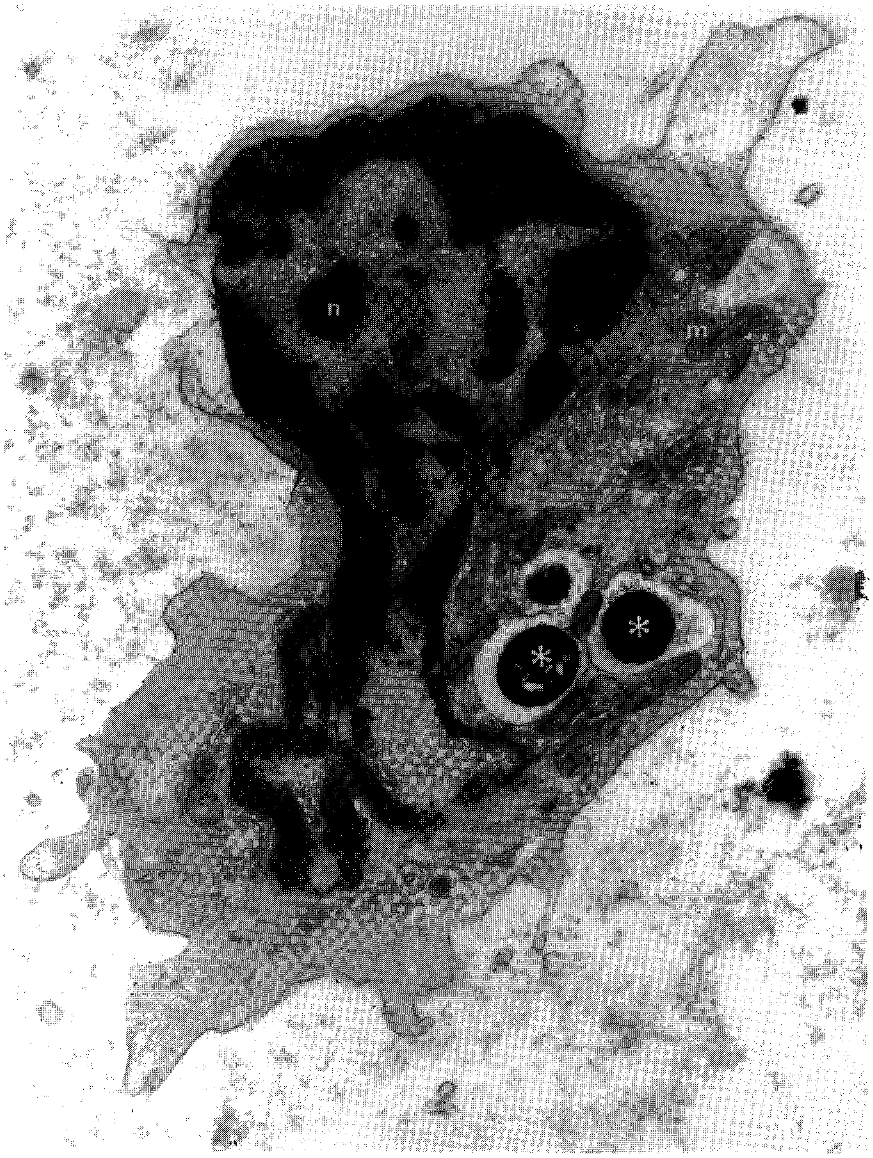

FIGURE 14. Electron micrograph of an isolated LGL labeled prior to embedding with Leu-19 antibody followed by gold colloid. There are 69 gold particles associated with the plasmalemma. Granules, asterisks; mitochondria, m; nucleus, n. ×22,000.

### DISCUSSION

In this study, we have characterized the phenotype and morphology of decidual LGL. Our immunoelectron microscopic (EM) findings have now provided definitive proof that cells with the morphology of what early European histopathologists called endometrial granulocytes or "K" cells (Weill, 1921; von Numers, 1953; Hamperl and Hellweg, 1958; Kazzaz, 1972) in the Rhesus monkey

FIGURE 13. (A) Electron micrograph of a characteristic large granular lymphocyte (LGL) in first-trimester decidua. There is a kidney-shaped-nucleus (n) short stubby cytoplasmic projections, long cisternae of rough endoplasmic reticulum (large arrowheads) mitochondria (M), and membrane-bounded granules (arrows). There is a portion of macrophage cytoplasm in the top left; compare the much smaller granules (small arrowheads) with those in the LGL. X8,800. (B) Electron micrograph of an LGL in first-trimester decidua. Note the granules of various sizes with (large arrowheads) and without (small arrowheads) a peripheral pale rim. Nucleus (n), mitochondria (m), golgi body (asterisk), glycogen (arrows). ×20,000. (C) Electron micrograph of a dividing LGL in first-trimester decidua. The cell type is identified by the three characteristic membrane-bounded granules (arrowheads). Chromosomes, c; endoplasmic reticulum, asterisks. ×12,000. (D) Electron micrograph of part of a gland (G) in first-trimester decidua which includes an intraepithelial LGL (asterisk) identified as such by its size, granules (arrows), and endoplasmic reticulum profiles (small arrowheads). There are numerous mitochondria (m) and lipid droplets (L) in the gland cells, which are separated from the connective tissue (t) by a basement membrane (large arrowheads). ×10,000.

(Cardell et al., 1969) and man (Jaeger and Dallenbach-Hellweg, 1969; Sengel and Stoebner, 1972; Loubet et al., 1989) are indeed CD56$^{+}$ LGL. Although this relationship was appreciated for some time (Bulmer and Sunderland, 1983; Bulmer et al., 1987), it had been difficult to confirm by light microscopy because the requirement of cryostat sections for immunostaining with Mabs, and in particular Leu-19 (CD56), also results in disruption of the granules by freezing. We find the EM morphology of decidual LGL very similar to that described for LGL in peripheral blood, although we have not observed the parallel tubular arrays, which are a consistent feature of the latter. These unique organelles are considered to be a typical structural characteristic of human NK cells (Huhn et al., 1982; Kang et al., 1988), so their absence in decidual CD56$^{+}$ LGL argues against these cells being classical NK cells.

We have analyzed the phenotypic characteristics of mechanically disaggregated decidual LGL both qualitatively and quantitatively using flow cytometry. Our qualitative observations using double immunostaining have confirmed that CD56$^{+}$ decidual LGL are CD3$^{+}$, CD16$^{dim+/-}$, and CD2$^{+}$. A previous flow cytometry study using enzymic disaggregation of decidual LGL noted an appreciable CD2$^{-}$ population (Starkey et al., 1988), whereas our present study, using mechanical disaggregation, finds only 5–10% of CD56$^{+}$ decidual LGL are CD2$^{-}$. It is possible that enzymic extraction could have resulted in the loss of certain surface antigens, particularly CD2, which is known to be vulnerable to proteolytic treatments (Ritson and Bulmer, 1987a). Our quantitative analyses have revealed that the density of CD56 on decidual LGL is very high, reaching a level 22× that found in the majority of CD56$^{+}$ PBL and 5× the level of the brightest CD56$^{+}$ PBL population. In contrast, the density of CD16 on decidual LGL is low or absent. Microscopic examination of Geimsa-stained preparations after cell sorting show that the majority of CD56$^{bright+}$ decidual LGL (75%) are granulated with a small number of agranular cells. This is in contrast to the CD56$^{bright+}$ subset in peripheral blood, which are mainly agranular (Lanier et al., 1986). Thus, decidual LGL are granulated cells with the phenotype CD56$^{bright+}$, CD3$^{-}$, and CD16$^{-}$. They differ from the CD56$^{bright+}$ PBL in being mainly granulated and expressing an increased density of CD56.

Functionally, we have previously observed that decidual LGL are responsive to the proliferative effects of IL-2 (100 U/ml) and are transformed by this lymphokine into potent lymphokine-activated killer (LAK) cells with marked NK cytolytic activity against susceptible targets, including normal and malignant trophoblast (King and Loke, 1990a). In contrast, naive decidual LGL show only moderate cytolytic activity (King et al., 1989b; Manaseki and Searle, 1989; Ferry et al., 1990). These functional properties are very similar to those reported for peripheral blood CD56$^{bright+}$ cells and are different from classical CD16$^{bright+}$ NK cells with respect to both the levels of cytotoxicity and the proliferative response to IL-2 (Nagler et al., 1989).

The phenotypic and functional characteristics of decidual LGL, therefore, correspond closely to those described by Lanier and his colleagues for a tiny subset of NK cells identified in peripheral blood. These investigators suggested recently that these small CD56$^{bright+}$ subsets may represent immature stages in the classical NK cell differentiation pathway, although they have not dismissed entirely the possibility that they represent distinctive NK/LGL subsets with varied functions (Nagler et al., 1989). We favor the second of these hypotheses and propose that the CD56$^{bright+}$, CD16$^{-/dim+}$ decidual LGL arise from a different, but closely related, LGL lineage rather than representing an early differentiation stage of classical NK cells. We have observed virtually no CD16$^{bright+}$ cells among decidual LGL, so there is no evidence of progressive acquisition of this surface molecule in decidua as there is thought to be in peripheral blood. Previous flow cytometric studies did report on the finding of up to 10% CD16$^{+}$ cells in decidua (Starkey et al., 1988; Manaseki and Searle 1989), but the fact that these cells were NKH-1 (CD56) negative and that the Mab used (Leu-11c) is not specific for the NK-cell Fc receptor would suggest that these cells are probably neutrophil granulocytes or possibly even trophoblast. Immunohistological staining of decidual sections also failed to reveal any CD16$^{+}$ cells (Ritson and Bulmer, 1987b; King et al., 1989a), reflecting our flow cytometric results of the small numbers of cells expressing CD16 at low density. Furthermore, albeit indirect support for our hypothesis comes from the observation that the staining of CD56 on decidual LGL is so much more intense than any of the peripheral blood CD56$^{+}$ populations and such CD56$^{bright+}$ cells have not been observed in any organ, or at any other mucosal surface (Mowat, 1990), besides the uterus.

The idea that uterine LGL are different from classical NK cells is given further support from work in mice. A recent immunohistological study has identified the granulated lymphoid cells in pregnant mouse uterus—the granulated metrial gland (GMG) cells—as perforin-containing NK-like cells that can be seen to proliferate *in utero* from small agranular precursors to large granular lymphocytes (Parr et al., 1990). Depletion of NK cells with asialo-GM1 antibody results in ablation of NK activity from the spleen, but NK function in decidua is only partially reduced (Croy et al., 1985). It has proved difficult to identify the cells responsible for NK activity in murine decidua because their isolation is difficult technically, but the putative candidates are thought to be the GMG cells (Croy, 1990). From her extensive studies, Croy has also been cautious in classifying GMG cells as classical NK cells. Recently, she crossed mice with no mature T or B cells (scid/scid) with those with defective NK function (bg/bg) and found that the resulting progeny reproduced normally, implying that neither T, B, nor NK cells were essential for reproduction. However, the typical giant lyzozomes found in all granulated cells of bg/bg mice, including NK cells were not identified in the uterine GMG cells, whose number and morphology were identical to GMG cells in normal mice (Croy and Chapeau, 1990). This finding, therefore, implies murine uterine granulated lymphocytes are distinct from the classical NK- or T-cell lineages.

Whichever hypothesis regarding the lineage of decidual LGL should eventually prove to be correct, the fact that these cells are so numerous (>70% of bone-marrow-derived cells) in decidua compared to peripheral blood (<1%) implies that there is some mechanism for selective recruitment to or inductive proliferation within the uterus. That the latter does occur *in vivo* is confirmed by our present observation of the nuclear proliferation marker Ki-67 in many $CD56^+$ decidual LGL. Previous studies have noted that cell proliferation in the endometrium continues throughout the menstrual cycle (Ferenczy et al., 1979). Recently, $CD45^+$ lymphoid cells were shown to be almost entirely responsible for this proliferative activity (Pace et al., 1989; Tabibzadeh 1990). We now demonstrate that this is attributable to the $CD56^+$ uterine LGL and that proliferation of LGL continues in early pregnancy. This finding may appear to contradict the old reports that mitotic figures were rarely, if ever, found in endometrial granulocytes (Hamperl and Hellweg, 1958). However, although we have noted mitoses in Giemsa-stained smears of isolated decidual and endometrial leukocytes, mitotic figures were not seen in granulated cells (unpublished). This suggests that the granules are not present in dividing cells and may appear *in utero* only as the LGL differentiate. Previous reports have stressed that because the numbers of endometrial granulocytes/LGL vary during the menstrual cycle, it seemed likely that they were subjected to hormonal control. Our present finding of a sex difference in the numbers of these LGL in peripheral blood of male and female individuals supports a role for hormones. It is of interest to note that LGL are found only in areas where stromal cells show evidence of decidual change either in the uterus or in ectopic foci of decidualization in the tube, cervix, or ovary (Hamperl and Hellweg, 1958). We have observed that decidual cells secrete significant amounts of laminin (Loke et al., 1989), the production of which is enhanced by progesterone (unpublished). IL-2-stimulated decidual LGL also produces laminin (unpublished). Perhaps homotypic interaction between LGL and decidual cells via this adhesion molecule can provide one mechanism for LGL homing and retention in the uterus.

Our present study also indicates that decidual LGL are in a state of activation different from $CD56^+$ LGL in peripheral blood. CD69 is absent from $CD56^{bright+}$ PBL, yet is present in decidual $CD56^+$ cells (Nagler et al., 1989). This antigen is induced rapidly on peripheral blood NK cells after IL-2 stimulation (Lanier et al., 1988). In addition, HML-1, originally thought to be confined to intestinal lymphocytes, is now thought to be an activation antigen as it can be induced on Con-A blasts from peripheral blood (Shieferdecker et al., 1990). HML-1 is also present on decidual LGL.

The CD56 molecule is identical to the embryonic form of the neural-cell adhesion molecule (E-NCAM) (Lanier et al., 1989), so the very high level of its expression on decidual LGL could function as an adhesion molecule regulating binding to other decidual elements, including the fetally derived placental trophoblast cells. We could demonstrate no conjugate formation between trophoblast and decidual LGL (King et al., 1990), which could be due to the highly sialylated molecules on both LGL (CD56/E-NCAM) and trophoblast (Whyte and Loke, 1978). Other adhesion molecules also expressed at high density on decidual $CD56^+$ cells include the integrins CD18, CD11a, and CD11c, and these might be important in migration and in determining the characteristic tissue distribution of LGL.

The functions of uterine LGL are not known and, in particular, the question of whether they are essential for pregnancy remains unanswered. It would be reasonable to suggest that an appropriate target for interaction is with the invading extravillous trophoblast cells of the placenta. NK cells produce many cytokines (Cuturi et al., 1989) and those produced are different in the various $CD56^+$ subsets (Nagler et al., 1989). Although there is no information on cytokine production by human decidual LGL, in the mouse, CSF-1, IL-1, and LIF have been identified in GMG-cell conditioned media (Croy et al., 1989). These cytokines may influence trophoblast growth, differentiation, and migration. From our observation that decidual $CD56^{bright+}$ cells are activated by IL-2 to become LAK cells capable of killing normal trophoblast and to an even greater extent malignant choriocarcinoma cells, we have proposed that decidual LGL could also control against undue invasion or malignant transformation (King and Loke, 1990b). A similar proposal has been made on the basis of murine studies (Head, 1989). The nature of these cellular interactions has not been established. There is now a great deal of interest in the nonclassical HLA-G antigen whose expression appears to be restricted only to extravillous trophoblast (Ellis et al., 1990; Kovats et al., 1990). It is tempting to suggest that this could be the putative target molecule recognized by decidual LGL. The expression of a non-polymorphic Class I molecule may explain the survival of trophoblast in the uterus. NK cells reject cells with absent or low Class I expression, whereas allotypic CTL will reject cells bearing classical Class I molecules (Storkus et al., 1989). This phenomenon has led to the "missing self" hypothesis, which states that the absence of self-HLA on target cells is seen by NK cells, rather than the presence of some other distinct NK receptor (Ljunggren and Karre, 1990). Trophoblast may have evolved a mechanism to escape attack by both allospecific T cell and NK cells by the expression of Class I HLA-G, which may be seen by the mother as self or neutral MHC. Interestingly, HLA-G is a relatively primitive antigen in phylogenetic terms (Watkins et al., 1990). We suggest that decidual LGL are also primitive lymphocytes that differentiate in the uterus along a separate pathway to that of classical NK cells, presumably as an adaptation to accommodate placentation in mammals. The idea that lymphocytes may differentiate extrathymically at mucosal surfaces is not new (Fichtelius, 1968; Campana et al., 1989). Analogous granulated $CD3^-$, $CD7^+$, but $CD56^-$ cells are found in the intraepithelial component of the gut (Jarry et al., 1990). Reproductive immunologists have traditionally considered the fetomaternal relationship primarily in terms of transplantation immunology, which is an artificial model in a highly evolved species. In the broader context of evolution, placentation in mammals must have developed a separate mechanism of immunological recognition based perhaps on some preexisting primitive defense system.

### MATERIALS AND METHODS

### Peripheral Blood Lymphocytes

Peripheral blood lymphocytes (PBL) from men and women aged 18–25 years sampled at the same time each day were isolated by centrifugation over Lymphoprep (Flow).

### Decidual Leukocytes

Decidual cell preparations were prepared as described previously (King et al., 1989a, 1989b). Briefly, pieces of decidua were obtained from first-trimester elective vaginal termination of pregnancy (6–12 weeks). The fragments of decidua were identified macroscopically and washed in RPMI. They were then minced between two scalpel blades and pushed through a 53 $\mu$m sieve (Gallenkamp). The cell suspension was washed, layered onto Lymphoprep, and spun at 400 g for 20 min. The cells were removed from the interface, washed, and resuspended at $3 \times 10^7$ cells/ml in phosphate buffered saline/0.1% bovine serum albumin (PBS/0.1% BSA).

### Culture of Decidual Leukocytes

Decidual leukocytes were cultured as previously described (King and Loke, 1990). Briefly, they were resuspended in RPMI and 10% fetal calf serum (FCS) supplemented with antibiotics and 2 mM L-Glutamine and 100-U/ml recombinant IL-2 (rIL-2) (Sigma). After incubation in 25-mm$^2$ flasks, the non-adherent cells were transferred to a new flask and cultured upright in 5% $CO_2$, 95% air at 37°C.

### Monoclonal Antibodies

The source and specificities of the monoclonal antibodies (Mabs) used in this study are shown in Table 2.

TABLE 2
Leukocyte Differentiation Antigens Identified by Monoclonal Antibodies Used in this Study

| CD cluster | mAb | Cellular reactivity | Source |
|---|---|---|---|
| CD2 | Leu-5b | T cells, NK cells | Becton Dickinson (BD) |
| CD3 | Leu-4 | T cells | BD |
| CD4 | Leu-3a | Helper/inducer T-cell subset | BD |
| CD5 | Leu-1 | T cells, B-cell subset | BD |
| CD7 | YTH 3.2.6 | T cells, NK cells | Prof. H. Waldmann[a] |
| CD8 | Leu-2a | T cells, NK-cell subset | BD |
| CD11a | YTH 81.5.1. | LFA-1 $\alpha$ chain | Prof. H. Waldmann[a] |
| CD11c | Leu-M5 | Monocytes, T-cell subset, NK cells | BD |
| CD16 | Leu-11b | NK cells, granulocytes | BD |
| CD18 | YFC 118.3.2 | LFA-1 $\beta$ chain | Prof. H. Waldmann[a] |
| CD45RA | 4KB5 | NK cells, T-cell subset, B cells | Dako |
| CD56 | Leu-19 | NCAM; NK cells, T-cell subset | BD |
| CD57 | Leu-7 | NK-cell subset, T-cell subset | BD |
| CD69 | Leu-23 | Activated T cells, B cells, NK cells | BD |
| | HLA-DR | Monocytes, activated T cells, B cells | BD |
| CD45 | Dako-LC | Leukocytes | Dako |
| | HML-1 | Intestinal lymphocytes | Dr. Cerf-Bensussan[b] |
| | Ki-67 | Nuclear proliferation antigen | Dako |

[a]All these mAbs were raised in rats and were gifts from Prof. H. Waldmann.
[b]Gift from Dr. Cerf-Bensussan.

### Immunofluorescence

All antibodies were diluted in PBS/0.1% BSA (PBS) and all incubations were conducted at 4°C.

(a) Direct Staining.

$1.5 \times 10^6$ cells were incubated with 50 $\mu$l of the first fluorescein- (FITC) or phycoerythrin- (PE) conjugated antibody for 30 min and washed twice in PBS. In single direct staining, the cells were then fixed with an equal volume of 2% formaldehyde (100 $\mu$l) and PBS. Samples were kept at 4°C in the dark until analyzed.

(b) Indirect Staining.

Following incubation with an unconjugated antibody, a secondary conjugated antibody, either goat antimouse FITC (GAM.FITC, Sigma) or rabbit antirat FITC (RAR.FITC, Sigma) was diluted in RPMI and 20% human AB serum (Sigma), incubated at 4°C for 30 min, and then spun 10 min at 200 g. 100 $\mu$l of this secondary FITC-conjugated antibody was added to the cells (30 min, 4°C), which were then washed and fixed as before.

(c) Double Staining.

An indirect immunofluorescence stain was followed by a direct stain. Thus, the cells were further incubated with 10–15-fold excess of purified mouse IgG (Sigma) for 10 min at 4°C. After washing, PE-conjugated Leu-19 was added for 30 min, 4°C, and washed and fixed as before.

(d) Controls.

Fluorochrome-conjugated isotype-matched irrelevant Mab IgG1+IgG2 (Simultest; Becton Dickinson) was used as a negative control. A positive control—Leu-4—was always used to confirm the effectiveness of GAM-FITC Ab and to quantify the accuracy of selective scatter gating. A positive dual fluorescence control was also used (Leukogate; Becton Dickinson). In addition, a negative control of GAM-FITC Ab alone was essential to set the threshold on the immunofluorescence histogram, above which the cells are considered positive for the Ab binding. In double staining, GAM-FITC Ab followed by an irrelevant directly conjugated Ab was used as the negative control.

The samples were analyzed on a FACStar Plus (Becton Dickinson).

### Histology

Samples of endometrium were taken from routine hysterectomy specimens as previously described (King et al., 1989a). Decidual and endometrial fragments (1 cm²) were frozen in liquid nitrogen for immunohistology. A piece was fixed in formalin for routine histology. The stage of the menstrual cycle was assessed as previously described (King et al., 1989a). Samples (2 mm) for electron microscopy were fixed for 1 hr in either 0.01 M periodate–0.075 M lysine–2% paraformaldehyde (PLP), or 1% glutaraldehyde with 3% paraformaldehyde in 0.01 M cacodylate buffer, then sorted in 0.1 M cacodylate buffer at 4°C until required. The tissues were postfixed for 1 hr in 2% osmium tetroxide with 1.5% potassium ferrocyanide buffered with veronal acetate at pH 7.2, dehydrated in increasing concentrations of ethanol followed by propylene oxide and embedded in Araldite. 75 $\mu$m sections were stained with uranylacetate and lead citrate prior to examination in a JEOL 100C electron microscope.

**Immunohistology**

A double immunohistological method was performed using a Mab to a nuclear proliferation marker (Ki-67) (Gerdes et al., 1984) and CD56 to identify uterine LGL. All incubations were performed in a wet box with two washes with tris buffered saline (TBS) in between incubations. 5 $\mu$m frozen sections were air dried, fixed in acetone (4°C, 5 min), rehydrated in TBS, and Ki-67, (1/10) applied to the section. A 30-min incubation with biotinylated antimouse antibody (1/100) (Dako) was followed by peroxidase-conjugated avidin (Dako) (1/400). This reaction was visualized using 3,3'diaminobenzidine (DAB) with 0.01% hydrogen peroxide. The sections were then incubated with normal mouse serum (1/10) (Sigma) for 10 min. Excess serum was wiped off the sections and Leu-19 (1/50) was applied for 30 min. Rabbit antimouse immunoglobulins (Dako) (1/20) in 10% human AB serum was then incubated for 30 min. After further washing, APAAP complexes (Dako) (1/50) were added for 30 min. The reaction was developed using a red substrate (Napthol AS-MX phosphate, dimethyl formamide, 200 ml, 9.8 ml of 0.1 M Tris-HCl buffer, pH 8.2, levamisole 3 mg, and fast Red TR sale 10 mg), which was stopped with tap water. After counterstaining in Carazzi's haemotoxylin for 5 min, the slides were mounted in glycerol gelatin (Sigma).

**Immunoelectron microscopy**

(a) Post-embedding Localization on Tissue Sections.

Tissue samples stored in 0.1 M cacodylate buffer were dehydrated in increasing concentration of ethanol and embedded either in Araldite at room temperature or in Lowicryl K4M at −20°C (Wells, 1985). These sections were incubated with Leu-19, or an irrelevant Mab, Leu-M3, which identifies macrophages. Both antibodies were diluted 1/10 in PBS +1% BSA +0.05% Thimerosal (Sigma). The sections were then treated with either (i) goat antimouse gold colloid (Janssen Pharmaceutica) (1/30); (ii) rabbit antimouse IgG (Dako) (1/100), followed by goat antirabbit gold colloid (Janssen Pharmaceutica) (1/30); (iii) rabbit antimouse IgG, followed by Protein A-coated gold colloid (Janssen Pharmaceutica). All incubations were carried out at room temperature for 40 min with two PBS washes between incubations.

(b) Pre-embedding Localization on Isolated Cells.

Isolated decidual cells were incubated with either Leu-19 or Leu-M3, cells were then fixed in suspension with PLP for 15 min, 4°C, washed twice, and resuspended in PBS. For the single-sandwich technique, the cells were resuspended in 80 $\mu$l of goat antimouse IgG adsorbed to 15-nm gold particles for 40 min at room temperature and then washed twice in PBS. For the double-sandwich methods, the cells were suspended in 80 $\mu$l of rabbit antimouse IgG (1/100), 60 min, and washed twice in PBS. They were then resuspended in 80 $\mu$l of goat antirabbit IgG adsorbed to 15-nm gold particles (1/25) for 40 min at room temperature and washed twice in PBS.

### ACKNOWLEDGMENTS

We acknowledge financial support from the Medical Research Council, East Anglian Regional Health Authority, the Wellcome Trust, and the Special Programme of Research, Development and Research Training in Human Reproduction, World Health Organization. We would also like to thank our obstetric colleagues and staff at Addenbrooke's Hospital for collecting the placental material.

(*Received November* 15, 1990)

(*Accepted November* 21, 1990)

### REFERENCES

Bensussan C., Jarry A., Brousse N., Lisowska-Grospierre B., Guy-Grand D., and Griscelli C. (1987). A monoclonal antibody (HML-1) defining a novel membrane molecule present on human intestinal lymphocytes. Eur. J. Immunol. **17**: 1279–1285

Bulmer J.N. (1989). Decidual cellular responses. Current Opinion Immunol. **1:** 1141–1147.

Bulmer J.N., Hollings D., and Ritson A. (1987). Immunocytochemical evidence that endometrial stromal granulocytes are granulated lymphocytes. J. Pathol. **153:** 281–288.

Bulmer J.N., Morrison L., and Smith J.C. (1988). Expression of Class II MHC gene products by macrophages in human uteroplacental tissue. Immunology **63:** 707–714.

Bulmer J.N., and Sunderland C.A. (1983). Bone-marrow origin of endometrial granulocytes in the early human placental bed. J. Reprod. Immunol. **5:** 383–387.

Campana D., Janossy G., Coustan-Smith E., Amlot P.L., Tian W.-T., Stephen I.P., and Wong L. (1989). The expression of T cell receptor-associated proteins during T cell ontogeny in man. J. Immunol. **142:** 57–66.

Cardell R.R., Hisan F.L., and Dawson A.B. (1969). The five structure of granular cells in the uterine endometrium of the rhesus monkey (*Macaca mulatta*) with a discussion of the possible function of these cells in relaxin secretion. Amer. J. Anat. **124:** 307–340.

Croy B.A. (1990). Granulated metrial gland cells—interesting cells found in the pregnant uterus. Am. J. Reprod. Immunol. **23:** 19–21.

Croy B.A., and Chapeau C. (1990). Evaluation of the pregnancy immunotropism hypothesis by assessment of the reproductive performance of young adult mice of genotype scid/scid. bg/bg. J. Reprod. Fertil. **88:** 231–239.

Croy B.A., Gambel P., Rossant J., and Wegmann T.G. (1985). Characterization of murine decidual natural killer (NK) cells and their relevance to the success of pregnancy. Cell. Immunol. **93:** 315–326.

Croy B.A., Stinchcomb D., Gough N.M., and Guilbert L.J. (1989). Cytokine production is a function of murine uterine granulated metrial gland (GMG) cells. Cytokine **1:** 102.

Cuturi M.C., Anegun I., Sherman F., London R., Clark S.C., Perussia B., and Trinchieri G. (1989). Production of haematopoietic colony-stimulating factors by human natural killer cells. J. Exp. Med. **169:** 569–583.

Ellis S.A., Palmer M.S., and McMichael A.J. (1990). Human trophoblast and the choriocarcinoma cell line BeWo express a truncated HLA Class I molecule. J. Immunol. **144:** 731–735.

Ferenczy A., Bertrand G., and Gelfand M.M. (1979). Proliferation kinetics of human endometrium during the normal menstrual cycle. Amer. J. Obstet. Gynecol. **133:** 859–867.

Ferry B.L., Starkey P.M., Sargent I.L., Watt G.M.O., Jackson M., and Redman C.W.G. (1990). Cell populations in the human early pregnancy decidua; natural killer activity and response to interleukin-2 of CD56-positive large granular lymphocytes. Immunology **70:** 446–452.

Fichtelius K.E. (1968). The gut epithelium—a first level lymphoid organ? Exp. Cell. Res. **49:** 87–104.

Gerdes J., Lemke H., Baisch H., Wacker H.H., Schwab U., and Stein H. (1984). Cell cycle analysis of a cell proliferation associated human nuclear antigen defined by the monoclonal antibody Ki-67. J. Immunol. **133:** 1710–1715.

Hamperl H., and Hellweg G. (1958). Granular endometrial cells. Obstet. Gynecol. **11:** 379–387.

Head J.R. (1989). Can trophoblast be killed by cytotoxic cells? *In vitro* evidence and *in vivo* possibilities. Amer. J. Reprod. Immunol. **20:** 100–105.

Huhn D., Huber C., and Gastl G. (1982). Large granular lymphocytes: morphological studies. Eur. J. Immunol. **12:** 985–988.

Jaeger J., and Dallenbach-Hellweg F. (1969). Elektronenkiroskopische Befunde an den endometrialen Kornchenzellan des Menschen. Gynaecologia **168:** 117–125.

Jarry A., Cerf-Bensussan N., Brousse N., Selz F., and Guy-Grand D. (1990). Subsets of CD3$^+$ (T cell receptor $\alpha/\beta$ or $\gamma/\delta$) and CD3 lymphocytes isolated from normal human gut epithelium display phenotypical features different from their counterparts in peripheral blood. Eur. J. Immunol. **20:** 1097–1103.

Kang Y.-H., Carl M., Grimley P.M., Serrate S., and Yaffe L. (1987). Immunoultrastructural studies of human NK cells: 1. Ultracytochemistry and comparison with T cell subsets. Anat. Rec. **217:** 274–289.

Kang Y-H., Carl M., and Watson L.P. (1988). Ultrastructural and functional effects of lipopolysaccharide and interleukin-2 on human NK cells. Scanning Microsc. **2:** 1567–1586.

Kazzaz B.A. (1972). Specific endometrial granular cells. A semi-quantitative study. Eur. J. Obstet. Gynecol. **3:** 77–84.

King A., Birkby C., and Loke Y.W. (1989b). Early human decidual cells exhibit NK activity against K562 cell line but not against first trimester trophoblast. Cell. Immunol. **118:** 337–344.

King A., Kalra P., and Loke Y.W. (1990). Human trophoblast cell resistance to decidual NK lysis is due to lack of NK target structure. Cell. Immunol. **127:** 230–237.

King A., and Loke Y.W. (1990a). Human trophoblast and JEG choriocarcinoma cells are sensitive to lysis by IL-2 stimulated decidual NK cells. Cell. Immunol. **129:** 435–448.

King A., and Loke Y.W. (1990b). Uterine large granular lymphocytes: a possible role in embryonic implantation? Amer. J. Obstet. Gynecol. **162:** 308–310.

King A., Wellings V., Gardner L., and Loke Y.W. (1989a). Immunocytochemical characterisation of the unusual large granular lymphocytes in human endometrium throughout the menstrual cycle. Human Immunol. **24:** 195–205.

Kovats S., Main E.K., Librach C., Stubblebine M., Fisher S.J., and DeMars R. (1990). A Class I antigen, HLA-G, expressed in human trophoblast. Science **248:** 220–223.

Lanier L.L., Buck D.W., Rhodes L., Ding A., Evans E., Barney C., and Phillips J.H. (1988). Interleukin 2 activation of natural killer cells rapidly induces the expression and phospharylation of the Leu-23 activation antigen. J. Exp. Med. **167:** 1572–1585.

Lanier L.L., Le A.M., Civin C.I., Loken M.R., and Phillips J.H. (1986). The relationship of CD16 (Leu-11) and Leu-19 (NKH-1) antigen expression on human peripheral blood NK cells and cytotoxic T lymphocytes. J. Immunol. **136:** 4480–4486.

Lanier L.L., Testi R., Bindl J., and Phillips J.H. (1989). Identity of Leu-19 (CD56) leucocyte differentiation antigen and neural cell adhesion molecule. J. Exp. Med. **169:** 2233–2238.

Ljunggren H.-G., and Karre K. (1990). In search of the "missing self": MHC molecules and NK cell recognition. Immunol. Today **11:** 237–244.

Loke Y.W., Gardner L., Burland K., and King A. (1989). Laminin in human trophoblast-decidua interaction. Human Reprod. **4:** 457–463.

Loubet A., Loubet R., Leboutet M.J., and Laures J.C. (1989). Les cellules granulenses de l'endometre. Ann. Pathol. **9:** 92–99.

Manaseki S., and Searle R.F. (1989). Natural killer (NK) cell activity of first trimester human decidua. Cell. Immunol. **121:** 166–173.

Mowat A.M. (1990). Human intraepithelial lymphocytes. Springer Semin. Immunopathol. **12:** 165–190.

Nagler A., Lanier L.L., Cwirla S., and Phillips J.H. (1989). Comparative studies of human FcRIII-positive and negative natural killer cells. J. Immunol. **143:** 3183–3191.

Pace D., Morrison L., and Bulmer J.N. (1989). Proliferative activity in endometrial stromal granulocytes throughout menstrual cycle and early pregnancy. J. Clin. Pathol. **42:** 35–39.

Parr E.L., Young L.H.Y., Parr M.B., and Young J.D.-E. (1990). Granulated metrial gland cells of pregnant mouse uterus are natural killer-like cells that contain perforin and serine esterases. J. Immunol. **145:** 2365–2372.

Polli N., Matutes E., Robinson D., and Catovsky D. (1987). Morphological heterogeneity of Leu7, Leu11 and OKM1 positive lymphocyte subsets: an ultrastructural study with the immunogold method. Clin. Exp. Immunol. **68:** 331–339.

Ritson A., and Bulmer J.N. (1987a). Extraction of leucocytes from human decidua. A comparison of dispersal techniques. J. Immunol. Meth. **104:** 231–236.

Ritson A., and Bulmer J.N. (1987b). Endometrial granulocytes in human decidua react with a natural killer (NK) cell marker, NKH1. Immunology **62:** 329-331.

Sengal A., and Stoebner P. (1972). Ultrastructure de l'endometre humaine normale. III. Les cellules K. Z. Zellforsch. **133:** 47–57.

Shieferdecker H.L., Ullrich R., Weiss-Breckwoldt A.N., Schwarting R., Stein H., Reicken E.-O., and Zietz M. (1990). The HML-1 antigen of intestinal lymphocytes is an activation antigen. J. Immunol. **144:** 2541–2549.

Starkey P.M., Sargent I.L., and Redman C.W.G. (1988). Cell populations in human early pregnancy decidua: characterisation and isolation of large granular lymphocytes by flow cytometry. Immunology **65:** 129–134.

Storkus W.J., Alexander J., Payne J.A., Dawson J.R., and Cresswell P. (1989). Reversal of natural killing susceptibility in target cells expressing transfected Class I HLA genes. Proc. Natl. Acad. Sci. USA **86:** 2361–2364.

Tabibzadeh S. (1990). Proliferative activity of lymphoid cells in human endometrium throughout the menstrual cycle. J. Clin. Endocrinol. Metab. **70:** 437–443.

von Numers C. (1953). On the specific granular cells (globular leukocytes) of the human endometrium. Acta Pathol. Microbiol. Scand. **33:** 250–256.

Watkins D.I., Chen Z.W., Hughes A.L., Evans M.G., Tedder T.F., and Letvin N.L. (1990). Evolution of the MHC Class I genes of a new world primate from ancestral homologues of human non-classical genes. Nature **346:** 60–62.

Weill P. (1921). Etudes sur les leucocytes. I. Les cellules granuleuses des muqueses intestinale et uterine. Arch. d'Anatomie Microscop. **17:** 77–82.

Wells B. (1985). Low temperature box and tissue handling device for immunostaining in electronmicroscopy. Micron. Microsc. Acta **16:** 49.

Whyte A., and Loke Y.W. (1978). Increased sialylation of surface glycopeptides of human trophoblast compared with fetal cells from the same conceptus. J. Exp. Med. **148:** 1087–1092.

